# Supplementary material for: The relationship between physicians’ self-kindness and professional fulfillment and the mediating role of personal resilience and work-home interference: A cross-sectional study
Source: PLoS One. 2023 Apr 24;18(4):e0284507. doi: 10.1371/journal.pone.0284507 (PMC10124859; doi:10.1371/journal.pone.0284507)
Supplement: S1 Appendix — Translated from Dutch to English. (DOCX) [file pone.0284507.s001.docx]

**Supplemental Digital Appendix 1**

**NVVC Survey on Cardiologists’ Occupational Well-being***

*[Translated from Dutch to English]*

**Sociodemographic characteristics^[[1]](#footnote-1)^**

| .1\| I agree to participate in this research.   - Yes |
| --- |
| .2\| Date of consent.   - “Dropdown with dates” |
| .3\| What is your sex?   - Male - Female - Other |
| .4\| What is your age?   - Younger than 36 years old - 36 – 45 years old - 46 – 55 years old - 56 – 65 years old - > 65 years old |
| .5\| What type of hospital are you currently working in?   - Independent sector treatment center - General hospital - Top clinical hospital - University hospital |

**Self-kindness**

| .1\| I try to be loving towards myself when I’m feeling emotional pain. |
| --- |
| .2\| When I’m going through a very hard time, I give myself the caring and tenderness I need. |
| .3\| I’m kind to myself when I’m experiencing suffering. |
| .4\| I’m tolerant of my own flaws and inadequacies. |
| .5\| I try to be understanding and patient towards those aspects of my personality I don't like. |

*This subscale was answered on a 5-point Likert scale:* Never; Sometimes; Regularly; Often; Always

**Individual resilience**

| .1\| I tend to bounce back quickly after hard times. |
| --- |
| .2\| I have a hard time making it through stressful events. |
| .3\| It does not take me long to recover from a stressful event. |
| .4\| It is hard for me to snap back when something bad happens. |
| .5\| I usually come through difficult times with little trouble. |
| .6\| I tend to take a long time to get over set-backs in my life. |

*This subscale was answered on a 5-point Likert scale:* Totally disagree; Disagree; Neutral; Agree; Totally agree

**Work-home interference**

*How often does it happen that…*

| .1\| You have trouble combining your work and private life?^[[2]](#footnote-2)^ |
| --- |
| .2\| You do not have the energy to engage in leisure activities with your spouse/family/friends because of your job? |
| .3\| You are irritable at home because your work is demanding? |
| .4\| You find it difficult to fulfil your domestic obligations because you are constantly thinking about your work? |
| .5\| You have to cancel appointments with your spouse/family/friends due to work-related commitments? |
| .6\| Your work schedule makes it difficult for you to fulfil your domestic obligations? |
| .7\| You have to work so hard that you do not have time for any of your hobbies? |
| .8\| Your work takes up time that you would have liked to spend with your spouse/family/friends? |
| .9\| Your work obligations make it difficult for you to feel relaxed at home? |

*This subscale was answered on a 4-point Likert scale:* Never; Sometimes; Often; Always

**Professional fulfillment**

| .1\| I feel happy at work. |
| --- |
| .2\| I feel worthwhile at work. |
| .3\| My work is satisfying to me. |
| .4\| I feel in control when dealing with difficult problems at work. |
| .5\| My work is meaningful to me. |
| .6\| I’m contributing professionally (e.g. patient care, teaching, research, and leadership) in the ways I value most. |

*This subscale was answered on a 5-point Likert scale:* Totally disagree; Disagree; Neutral; Agree; Totally agree

**This study was part of a larger project about Dutch cardiologists’ occupational well-being. This appendix only shows the survey items/ variables relevant for our study. For an outline of the full survey including all items/ variables please contact the authors.*

1. Respondents could only select one answer option per sociodemographic item. [↑](#footnote-ref-1)
2. This first item differs from the first item in the official negative Work-Home Interference subscale of the Survey Work-Home Interaction – Nijmegen: “You do not fully enjoy the company of your spouse/family/friends because you worry about your work?”. The authors decided to change this item after thorough discussions with the Netherlands Society of Cardiology, because all felt that the “new” item covers more than the official item and would offer a more realistic picture of the (potential) work-home interferences Dutch cardiologists experience. [↑](#footnote-ref-2)
