# Supplementary material for: The relationship between physicians’ self-kindness and professional fulfillment and the mediating role of personal resilience and work-home interference: A cross-sectional study
Source: PLoS One. 2023 Apr 24;18(4):e0284507. doi: 10.1371/journal.pone.0284507 (PMC10124859; doi:10.1371/journal.pone.0284507)
Supplement: Attachment — Submitted filename: Response to Reviewers.docx [file pone.0284507.s002.docx]

Dear Editor,

We appreciate the valuable comments of the reviewers on our paper ‘The relationship between physicians’ self-kindness and professional fulfillment and the mediating role of personal resilience and work-home interference: A cross-sectional study’. We have studied the comments thoroughly and are pleased to provide you with the following answers and corresponding changes in our paper.

Also on behalf of the co-authors,

Rosa Bogerd

| **Editor/ reviewer(s)' Comments** | **Our response** | **Revisions (location of revisions in clean manuscript)** |
| --- | --- | --- |
| **Editor** |  |  |
| 1. Please ensure that your manuscript meets PLOS ONE's style requirements, including those for file naming. The PLOS ONE style templates can be found at {links}. | 1. Thank you for providing us with the links to the formatting templates. We adjusted the manuscript and title page accordingly. |  |
| 2. Please provide additional details regarding participant consent. In the ethics statement in the Methods and online submission information, please ensure that you have specified what type you obtained (for instance, written or verbal, and if verbal, how it was documented and witnessed). If your study included minors, state whether you obtained consent from parents or guardians. If the need for consent was waived by the ethics committee, please include this information. | 2. Information on participant consent was adjusted in the Ethical approval in the Methods. We have updated this information also in the online submission information:  “The institutional ethical review board of the Amsterdam UMC *(METC)* of the University of Amsterdam provided a waiver declaring the Medical Research Involving Human Subjects Act (WMO) did not apply to the current study (reference number W20_324 # 20.323). *Written informed consent was obtained in the online survey*. Participants were only allowed to continue the survey when they entered 'Yes' for "I agree to participate in this study". Information about the research was sent to the participants via email previously and again provided at the beginning of the survey.” | 2. – Line 233-237 |
| 3. Thank you for stating the following in the Competing Interests section (…). Please confirm that this does not alter your adherence to all PLOS ONE policies on sharing data and materials, by including the following statement: "This does not alter our adherence to  PLOS ONE policies on sharing data and materials.” (as detailed online in our guide for authors). If there are restrictions on sharing of data and/or materials, please state these. Please note that we cannot proceed with consideration of your article until this information has been declared. Please respond by return email with your amended Competing Interests Statement and we will change the online submission form on your behalf. | 3. We emailed our adjusted Competed Interests. The editorial assistant instructed us to include them in the revised cover letter. We did so and include them here as well:   *“I have read the journal's policy and the authors of this manuscript have the following competing interests: co-author Debby G. Keuken is a senior policy officer at the NVVC. Co-author Rutger J. Hassink was chair of the Quality Committee of the NVVC during the period of data-collection. Co-author José P. S. Henriques is a board member of the NVVC. The NVVC commissioned this project and was a party in the data agreements. In agreement with the NVVC, the authors have decided to make the data available upon request. The other authors have no conflicts of interest.”* |  |
| 4. We note that you have indicated that data from this study are available upon request. PLOS only allows data to be available upon request if there are legal or ethical restrictions on sharing data publicly. For more information on unacceptable data access restrictions, please see {link}.  In your revised cover letter, please address the following prompts:  a) If there are ethical or legal restrictions on sharing a de-identified data set, please explain them in detail (e.g., data contain potentially sensitive information, data are owned by a third-party organization, etc.) and who has imposed them (e.g., an ethics committee). Please also provide contact information for a data access committee, ethics committee, or other institutional body to which data requests may be sent.  b) If there are no restrictions, please upload the minimal anonymized data set necessary to replicate your study findings as either Supporting Information files or to a stable, public repository and provide us with the relevant URLs, DOIs, or accession numbers. For a list of acceptable repositories, please see {link}.  We will update your Data Availability statement on your behalf to reflect the information you provide. | 4. We have adjusted our Data Availability Statement accordingly (please also see our revised cover letter for this statement):  “The data underlying this article are partly owned by the NVVC. Predetermined agreements in the contract between both parties create legal restrictions on sharing the data. Data will be shared on reasonable request to the NVVC (bureau@nvvc.nl) or to the corresponding author (r.bogerd@amsterdamumc.nl) with permission of the Netherlands Society of Cardiology.” |  |
| **Reviewer 1** |  |  |
| 1. Abstract The first sentence of the conclusion overstates the results of this study, please revise. | 1. We thank the reviewer and agree that we have phrased the first sentence of our conclusion in the abstract too strongly. We rephrased it in order to most accurately reflect the findings of this study. We have substituted the words “indirectly enhances” for “may enhance”. | 1. Line 54 (Abstract conclusion) |
| 2. Introduction Page 4, line 93, “ant” should be “and”. | 2. We would like to thank the reviewer for noticing and corrected this typo. | 2. Line 89 (Introduction) |
| 3. Methods 1. Please describe how the sample size is calculated, and what the inclusion and exclusion criteria are. 2. I wonder the criteria the author included demographic characteristics, what was the reason to select sex, age, years of experience as a cardiologist (year of first registration) and type of institution? 3. Without univariate analysis, why the author only included gender and years of experience as covariates from 4 demographic characteristics? | 3. We thank the reviewer for the issues raised in the method section and are grateful for the opportunity to better explain and legitimize our choices made. We will deliberately respond to each separate point raised by the reviewer.  1. - Since we were interested in all Dutch practising cardiologists we invited all cardiologists registered as a member of the Netherlands Society of Cardiologists (NVVC) and practicing in the Netherlands for this survey study. No other in- or exclusion criteria were employed. The NVVC provided us with the email-addresses of all registered members practicing in the Netherlands. The survey invitation clearly mentioned that the research was commissioned by the NVVC. With this data-collection technique we had no reason to expect a different or deviant representation of the population in our sample. The demographic characteristics of our sample resemble the demographic characteristics of all NVVC members.  We have clarified the inclusion criteria in the method section in two places. In the “Study design, population and setting” section: “This cross-sectional survey study was conducted in 2020 amongst all registered cardiologists who were a member of the Netherlands Society of Cardiology (NVVC) *and were working in the Netherlands*.**”** and in the “Data collection” section we clarified that we: “invited all cardiologists *practicing* in the Netherlands *and* registered as an NVVC member.” Lastly, we added the following part to the “Results” section: *“The results are not too deviant from the average age of cardiologists in the Netherlands (almost 70% of our sample was between 36 and 55 years old) and the percentage of male cardiologists (72.0%); for type of hospital and years of experience no population numbers were available.”*  - To assess our sample we made use of Green’s (1991) general rule of thumb for determining sample sizes when testing individual predictors in regression analyses (which are used to build parallel mediation models): 104 + number of independent variables. As we used 5 independent variables in our model, the minimum required sample size would be 109 (104 + 5). Our sample size of 374 thus meets this criterion implying that it has enough power to detect existing relationships. We expected to be able meet this rule of thumb before we started the data collection, which is why we did not perform a power analysis a priori. Refs:  *Green SB. How many subjects does it take to do a regression analysis. Multivariate behavioral research. 1991;26(3):499-510. VanVoorhis CW, Morgan BL. Understanding power and rules of thumb for determining sample sizes. Tutorials in quantitative methods for psychology. 2007;3(2):43-50.*  We added the following explanation in the “Statistical analyses” section to describe how we assessed our sample size: *“We rightly expected that our data collection would result in a sample that exceeds the general rule of thumb for minimum sample sizes in regression analyses (of which our parallel mediation model consists). (Green, 1991, VanVoorhis & Morgan, 2007)” Therefore, we would have sufficient power to detect significant associations between the variables under study and we did not perform an a priori power analysis.”*  2. We chose to include the basic demographic characteristics sex, age, years of experience as a cardiologist, and type of institution in our questionnaire as these were deemed most relevant, and indeed most often used in studies into investigating physician occupational well-being.  We added this clarifying sentence to illustrate our decision in the manuscript: *“These four demographics are able to provide a basic description of our sample and were considered most relevant in explaining physicians’ occupational wellbeing by the researchers. Additionally, the NVVC deemed them potentially relevant for defining new, tailored wellbeing policies or interventions.”*  3. We understand the reviewer’s confusion at this point and we appreciate the opportunity to clarify our choices here. Years of experience and age were highly correlated (Pearson’s R = -.824, p < 0.001), meaning that their effect on our outcome variable professional fulfillment was largely comparable. Due to its clinical relevance, we therefore decided to only include years of experience as a covariate. Although not properly reported in our previous manuscript, we did perform univariate analyses (T-tests and ANOVA’s) for sex, age, years of registration and type of institution on personal resilience, work-home interference and professional fulfillment. No differences were found in outcomes for any of the demographic variables. However, as sex and years of registration are considered basic covariates used in physician well-being studies and we find that the journal’s readership may expect them, we did include them as covariates in our model.  We have expanded our method section as follows: *“Preliminary univariate analyses (T-tests and ANOVA’s) found no statistically significant relationship of any of the demographic variables with professional fulfillment. In line with other well-being research, however, sex and years of registration were included as covariates. Because of the multicollinearity of age and years of registration, we opted to use the latter in the analysis as it was more clinically relevant.”* | 1. - Line 127-129 (Study design, population and setting),     line 177-178 (Data collection),     line 243-246 (Results)  - Line 197-201 (Statistical analyses)    2. - Line 185-188 (Data collection)    3. - Line 220-224 (Statistical analyses) |
| 4. Results 1. In Table 1 and Table 2, the bottom lines were missed. 2. Is there any difference in professional fulfillment by demographic characteristics? | 4.1 We thank the reviewer for noticing this. We added bottom lines to Table 1 and Table 2.  4.2 We understand the reviewer’s curiosity. Preliminary univariate analyses (T-tests and ANOVA’s) found no differences in professional fulfillment by demographic characteristics. As this was not a research question, we did not report these findings initially. However, the reviewer made us realize that readers may be interested in this information. As was explained in our response to comment 3.3, in our method section we now describe that no statistically significant differences were found by any of the demographic characteristics. | 4.1 Page 12 & 13 (Results)  4.2 Line 220-225 (Statistical analyses) |
| 5. Discussion 1. The short titles are not appropriate. 2. The dependent variable is professional fulfillment, why the author focus on the score for self-kindness, not the score for professional fulfillment? 3. the discussion is need to be expanded deeply. | 5.1 In line with the reviewer’s comment we adjusted the short titles into: Main findings, Explanation of findings, Strengths and limitations and Implications for practice and research.  5.2 We are grateful for this comment and we agree that we should better clarify the professional fulfillment score of Dutch cardiologists. We have added a paragraph at the beginning of the discussion focusing on professional fulfilment:  *“The average professional fulfillment score of Dutch cardiologists (M = 3.85) is relatively high, compared to those of physicians in general which (converted to our measurement scale) have been reported between 3.25 and 3.50 (24, 53). One explanation for this finding may lie in the highly rewarding and meaningful nature of the specialty – cardiologists are frequently present at important moments in patients’ lives which may be a stimulator for their feelings of intrinsic rewards and motivation.(1, 39, 54) Factors that have been found to positively affect the professional fulfillment of Dutch cardiologists are indeed satisfying physician-patient interactions, but also autonomy, personal resilience, satisfaction with compensation levels and the degree to which the job matches an individual’s energy resources.(54) This study adds to that by showing how self-kindness indirectly affects cardiologists’ professional fulfillment. Previous research has shown that patients of professionally fulfilled physicians are more satisfied(1) and that self-kindness has been found to be a prerequisite for compassionate care (18, 55). Therefore, enhancing cardiologists’ self-kindness may benefit patients in multiple ways.”*  3. Thank you for the suggestion to deepen our discussion. To this end, we made to following adjustments:  - Adding one paragraph on professional fulfillment at the beginning of the discussion (please see our response to 5.2 above).  - Throughout the discussion section we added an emphasis on the relevance of self-kindness and professional fulfillment for patient care.  - We elaborated on the found association between self-kindness and personal resilience:  *“Research on physician resilience often stresses the influence of genetics, personality traits and an individual’s (professional) environment on one’s resilience(59, 60) – at times neglecting the possibility that trainable skills or attitudes may also boost individuals’ resilience. Our results add to the body of literature(61-63) which demonstrates that trainable skills, such as self-kindness, may also enhance physician resilience.”* | 5.1 Page 14-19 (Discussion)  5.2 Line 290-304 (Explanation of findings)    5.3 - Line 290-304 (Explanation of findings)  - Line 301-304, line 320-322, line 342-343 (Explanation of findings)  - Line 346-350 Explanation of findings) |
| **Reviewer II** |  |  |
| 1. The introduction can be improved. Explicitly introduce research questions / research objectives towards the end of the introduction, as bullet points, before sharing how the remaining article is structured. | 1. Thank you for your suggestions. In line with the reviewer’s comments we have made the following adjustments to the introduction:  - We adjusted our aims into research questions:  *“This study aims to contribute to the literature and practice by addressing the following research questions: To what extent 1) is cardiologists’ self-kindness related to their professional fulfillment? and 2) is this relationship mediated by personal resilience and work-home interference?”*  - We added a sentence at the end of our introduction sharing how the remaining article is structured:  *“In the next section we discuss our research methods, followed by the presentation of our results, a discussion of our findings and a conclusion.”* | 1. - Line 113-116 (Introduction)  - Line 122-123 (Introduction) |
| 2. The introduction must include the importance of this work can be highlighted at the end of the introduction. Also, the novelty of this paper should be further justified by highlighting main contributions to the existing literature. The authors need to provide a strong argument here to show their novelty contributed to the field, the entire readership and the research community. | 2. We thank the reviewer for the opportunity to further strengthen the importance and novelty of this study.  - We have highlighted the importance of our work at the end of our introduction by articulating the need for transforming the unhealthy and indeed detrimental aspects of medicine’s culture:  *“The outcomes of this study provide more insight into cardiologists’ attitudes towards the self and how they relate to their occupational well-being. Taking into account the aforementioned detrimental aspects of medicine’s professional culture, a better understanding of the role and impact of self-kind attitudes may offer a first step in finding ways to redefine and improve practice. (6, 7). The results of this study may inform new approaches to enhance physicians’ professional fulfillment and, subsequently, patient care.”*  - We have further justified the novelty of our paper by adding a clear gap to the second paragraph of the introduction, endorsing the novelty of our work. We argue that our paper contributes to the field, readership and research community because it adds evidence on how self-attitudes may come into play with regards to physician well-being in a positive manner instead of negative:  *“Previous research has shown how physicians’ self-critical and perfectionistic attitudes may harm their professional fulfillment (6, 7), but research on how opposite self-attitudes, such as self-kindness, may actually benefit it, is scarce. Empirical research on self-kindness as a potential predictor for professional fulfillment will add to the body of literature on physician well-being from a more positive angle.”* | 2. - Line 116-122 (Introduction)    - Line 90-94 (Introduction) |
| 3. In the methodology and research design section please define the population of the study and sampling technique adopted. Also please justify why your chosen sampling technique and sample size are appropriate. | We thank the reviewer for this clarifying question. As reviewer 1 also addressed these issues we have answered these comments above in our responses to comment #3.1 from reviewer 1. |  |
| 4. The authors are suggested to have a stronger discussion on the findings before the conclusion section. Discussion should ideally have 2-3 subsections on Contributions to literature, Implications for practice and Limitations and future research directions. | Thank you for providing us the opportunity to have a stronger discussion on the findings. We would like to refer this reviewer to our answers to comment #5 by reviewer 1 for the adjustments made in the discussion section. |  |
